# Economy

**Published:** 1883-03

**Authors:** 


### ECONOMY.

THERE is no greater error than to confound the idea of economy with stinginess ; and yet very many persons make this mistake.

What we call success in life, as regards the accumulation of wealth, always depends on habits of industry and of economy. Competencies are accumulated from what is saved after making reasonable provision for the requirements of life, such provision depending in a great measure on the social position and intelligence of the individual. Thrift and economy go together ; they command the respect even of those who possess neither. But there is a true economy, and there is a mistaken economy which amounts to wastefulness ; and he who knows just where to draw the line between them is on the road to fortune.

The true economist uses his time and his opportunities to the best advantage. He not only economizes his time but his health and his strength, knowing well that success depends on these. Mistaken economy wastes the "golden sands of time" in picking up bent pins and worthless scraps of iron.

There is a good deal of nonsense in some of those golden rules that a more reverent generation were wont to confound with sacred teachings. "The early bird that caught the worm" was long ago punished for his cruelty ; the man who "gathered up all the shreds of time and wove them in the cloth of life" has passed away. It has since been discovered that men need all the sleep that nature demands, and all the wholesome food that nature requires ; and that after these wants are supplied there is ample time for useful and profitable work in the active duties of life.

